# Selections and Abstracts

**Published:** 1900-11

**Authors:** 


### SELECTIONS AND ABSTRACTS.

### The General Therapeutical Action of Electricity.*

The use of electricity in medicine has become so thoroughly popularized that it is the exception to find a practitioner without some form of electrical appliance, and yet there seems to be in general a very indefinite appreciation of the effects of the passage of the current through living tissue, upon which are based the rationale of every electro-therapeutical result. The activity that prevails in this fascinating field of research is too frequently an activity without insight—an activity guided by no true appreciation of the laws which govern and the principles which underlie the subject. Since the writer, in conjunction with the late Dr. George M. Beard, enunciated those ideas in regard to the general therapeutical action of electricity which gave the first efficient impulse to electro-medical and electro-surgical investigation in this country, the literature of the subject has accumulated beyond measure, and yet so little correct understanding is there of its physics that it is necessary to emphasize the statement again and again that electricity is not a simple, single manifestation of natural force, but is a generic term including complex phenomena and a wide variety of manifestations.

To understand its physical and physiological laws is to appreciate the fact that it is a many-edged weapon.

Is it a stimulant? Undoubtedly it is a stimulant of the most powerful character, and an ugly irritant as well when this side of its nature is sought after. Is it a sedative? As a sedative it is in some cases unequalled, and can generally be relied upon to soothe in greater or less degree a functional hyperesthetic condition of the nervous system. Whether the word "tonic" should be dismissed from the vocabulary of medicine, as some have asserted, calls, it seems to me, for very little serious discussion.

*By A. D. Rockwell, A M., M.D., New York, in a "System of Practical Therapeutics." Lea Brothers & Co., Phila., 1900.

The derivation of the term from the Greek *teino*, "I stretch," and from still another word, *tontho*, "I brace or give vigor," is sufficiently suggestive. We speak of toning a violin, so that its strings give forth a clearer and better sound. We do this by making them more tense. By this figurative allusion our treatises on materia medica and therapeutics describe tonic medicines as those which gradually produce the requisite degree of tension of the nervous system and of the living fiber generally, and which enable it fitly to respond to all of its natural and appropriate stimuli. "The idea of tension is inseparably connected with all our notions of vital force, because the most common, if not the only, conception we possess of organic power is derived from our experience of the phenomena of muscular force, which is always displayed in connection with the tension of muscular fiber."

Admitting, then, the recognized significance of the word "tonic," and that remedies for improving the tone of the system still hold an important position in the classification of the materia medica, in my judgment electricity must be classed as a tonic of a very high order. It possesses varied influences of this kind, according to the kind of current used and the method of its application. To produce that tension of the nervous system and of the muscular fiber generally, so as to enable them to respond to their natural stimuli, the mechanical effects of the faradic current seems especially adapted. It is a current of alternation, of to-and-fro motion, of constant closing and breaking. Unlike the galvanic current, it possesses no chemical action to be feared, and no powerful reflex effects need render us unduly cautious in its use. In the passage through the body of a current interrupted with the requisite degree of rapidity it need produce no appreciable muscular contractions, but yet it gives passive exercise to all the deeper-lying as well as superficial tissues. The numerous branch currents going to and fro act as so many shuttlecocks, keeping every atom in incessant disturbance.

Aside from these purely mechanical effects, the experience of every electro-therapeutist must teach him that even the induced or faradic current, and especially currents of high potential and high frequency, as obtained from the Ruhmkorff coil and the Holtz influence machine, exert some other and more subtile influence upon the nerves themselves, for by no purely mechanical effect can we account for the numerous phenomena that follow their use in diseased conditions.

While these mechanical effects of electricity are constantly observed after simple local applications, yet he who uses these forms of electricity in local pathological conditions only, and exclusively for their local effects, will fall far short of eliciting their full therapeutic power. It is this appreciation of the broad therapeutic action of electricity, and a knowledge of the details of the general methods of application necessary for its production, that have aided in the success of so many charlatans. They have accomplished results along certain lines far more satisfactory than those of the scientific physician, who has restricted his excursions in electro-therapy to purely localized methods of application.

Electricity, then, after the method of general electrization, may be said to be indicated wherever a constitutional tonic influence is called for. This law renders the remedy as valuable to the general practitioner as to the specialist. For the tedious period of convalescence that often follows typhoid and typho-malarial fevers there is no remedy that is comparable to it, and in a large number of conditions of depressed vitality, electricity, in some one of its forms and by some one of its methods of application, will prove of more or less service. It gives passive exercise to the muscles, it promotes and renders more natural the processes of excretion and secretion; in a word, it imparts tone and strength to both nerve and muscle. With these general principles before us we cease to wonder that electricity is used and recommended for such a variety of diseases, many of them of an apparently opposite character, and we see the injustice of that criticism which condemns electricity because it is claimed to be of service in so many different conditions. When the therapeutic efficiency of electricity is denied, the inevitable conclusion at which those must arrive who have time and again witnessed evidences of its power is, that he who thus fails in his results is deficient in his experience, is not sufficiently persistent, or has somehow failed in the technique of his applications.

Some of the results in electro-therapeutics are due in a measure —although not in great measure, I apprehend—to physical effects. The heat excited in the tissues by the passage of either form of current is undoubtedly small, but, judging from analogy as well as facts, there can be little question but that an appreciable degree of heat is excited. All conductors of electricity become heated more or less according to the degree of their resistance, and as the body offers great resistance a certain proportion of the electric force must be converted into heat. It is, at all events, a demonstrated fact that cold extremities are sensibly warmed by currents of high frequency and high potential, even when but few muscular contractions are produced.

Electricity possesses well-known physical effects on metals. Platinum wires are contracted by the passage of electric currents through them, and copper wires that are freely used for conductors become brittle, and it is said that the copper wires that conduct faradic currents break more speedily and more frequently than those which conduct galvanic currents. These physical facts are very suggestive of what may be facts in physiology and pathology sufficient to account for some of the therapeutic effects of electrization that as yet have hardly emerged from the realm of empiricism. These possible physical and physiological changes apply especially to the after or secondary effects of electrical treatment—effects that are noticed, not while the applications are being made or during the course of the treatment, but weeks and months after the treatment has been discontinued.

The chemical or electrolytic influences that are associated with electrical action are practically confined to the galvanic current. Its visible effects occur when needles or pointed electrodes of metal are used, and its especial utility is in the department of electro-surgery, although it is undoubtedly true that some electrolytic action occurs in all ordinary applications of the galvanic current to the body whatever form of electrode is used, and is probably a factor of more or less efficiency in making up the sum of electro-therapeutical effects.

A distinction has been made, and justly, between electrolysis and galvano-chemical cauterization. The one is a disintegration and separation of the constituent elements of organized structure. The other produces its effects by means of the acids and alkalies that are liberated at either pole by electrolytic action. It is the chemical galvano-cauterization, and not the electrolysis, that accomplishes most of the good results that follow intra-uterine applications in cases of endometritis, and yet that the beneficial effects are not due solely to the acids and alkalies is evident from the fact that when ordinary acids and alkalies are applied to the body, eschars of the same degree and kind are not obtained. The current penetrates and pervades the tissues, and induces various changes beyond and beneath the eschar which continue long after the current ceases to flow.

In its influence over nutrition, however, the physiological effects of electricity are perhaps the most important. Mechanical, physical, and chemical effects must indeed combine to make up physiological effects. The former are observed on dead as well as living, on inorganic as well as organic substances; the latter are peculiar to the living body only. Physiological effects are those which take place by virtue of the vital properties of the body, and cease when life ceases. The action of all currents modifies physiological function in various ways.

First. It may increase it. This action of the galvanic current is so well known as hardly to need mention. The readiness with which the secretion of the salivary gland can be increased is readily demonstrated. Mucous secretions are very quickly increased by the action of either current, and the effects of electrization are being utilized more and more in diseases characterized by a suppression of the normal mucous secretions. Especially in the so-called dry catarrh is the treatment efficacious, as has been shown in many cases. The effect of electricity on the biliary secretion, on the gastric juice, and on the intestinal fluid, is probably stimulating, but is less easy of mathematical demonstration. Certainly, these fluids ought to be secreted in greater abundance under the influence of the current, and the results of treatment in pathological cases give this probability something of the force of certainty. My own experience is that the appetite is sharpened, digestion quickened, and constipation relieved both by local and general electrical treatment

—so decidedly in many cases as to make it pretty evident that the gastric and intestinal glands are made to secrete more liberally by the action of the current upon the nerves that suppply these organs than on the tissues of the organs themselves. These results, that have been familiar to me for years, have recently been confirmed by Ziemssen in a very interesting manner by experiments on animals. He found that either form of current would increase the secretion of the various glands.

The subjective effects of the electric treatment act the same way in the bowel as in the stomach, by invigorating and improving the appetite. In the bowel it diminishes or removes the painful tenderness in the lower part of the body, as well as the feeling of movement or heat that is frequently experienced. The objective effects of the treatment, Ziemssen conjectures to be, that the stimulation of the motor nerve excites the intestinal ganglia, and thereby increases the flow of secretion from the intestinal glands, as well as induces a flow of the pancreatic and biliary secretion.

Among those affections of the bowels in which electricity is quite certain to exercise a beneficial influence are defective innervation, atrophy of the muscles of the bowels, digestive atony, and that large class of diseases comprehended under the name hypochondriacal and neurasthenic.

Second. Electricity may not only diminish, but it may arrest, the physiological activity of the organs of secretion and excretion.

When, as in polyuria, the general application of the current is often followed by a marked diminution in the flow of urine, it is a desirable therapeutic effect, but the effect to which I allude is far from desirable. It comes from the use of currents so strong as to paralyze glandular function. On the lacteal secretion it acts with decided though varying power in increasing it, or in restoring it after it has been temporarily suppressed; but in two cases I have known this secretion to be promptly suppressed by the injudicious use of currents of high tension. On the circulation the influence of electricity is a complex study, since it comprehends its action on the heart and on the unstriped muscular fibers of the arteries, as well as upon the central and peripheral nervous system. Certainly, very sensible modifications of the circulation may be observed in any strong and prolonged application of either current, and generally on the side of an increase of the flow of blood and some elevation of temperature, with dilatation of the veins.

On the absorbents the action of electricity has been found to be of practical value in relieving many hypertrophies, effusions, and morbid growths. To follow out the rationale of its operation in producing these effects would be a difficult and tedious task, and it can only be said here that the effects of electricity on the excretory processes of the body, its direct chemical action, and its influence over the circulation in the arteries, veins, and capillaries by its power to increase the quantity and quality of innervation received by them must constitute most important factors in influencing the process of absorption.

Not only does electricity directly influence nervous action through the vaso-motor system, but it excites vital function by acting directly on the cell itself and on the protoplasm, thus hastening nutritive changes and cellular activity; excretion is stimulated and poisons are eliminated, thus rendering it invaluable in the treatment of rheumatic conditions.

In further explanation of the rationale of the therapeutic action of electricity, the discovery of M. Edouard Branley,* if not conclusive, is at least most interesting, and may throw light on the pathology and treatment of the functional neuroses. Through no possible interpretation could it have interest for us on the old theory of the anatomical continuity of the nervous system; but in the light of the discovery of the neuron, by which we find the nerve tissue made up of distinct and disconnected elements, it immediately becomes worthy of consideration. Briefly stated, M. Branly discovered that if a tube of iron-filings be placed in a weak galvanic circuit the meter fails to register, thus showing that the substance is practically non-conducting. If, however, the tube is previously placed in a solenoid through which course currents of high frequency, or is simply subjected to the influence of static electricity or of the cathodic ray, the tube instantly becomes a conductor and the current passes. Subject the tube with its contents,

*See *L'Actualite Medicale*, December 15, 1897. *Revue Internationale d'E'ectrotherapie*, March, April and May, 1898.

however, to some sudden shock, as that of a static spark or blow, and its conductibility is immediately annihilated. Various theoretical considerations, unnecessary to discuss here, are advanced in explanation of these phenomena. They may or may not be the true solution of this mysterious happening; but we certainly see a similarity between the individual iron-filings of the tube and the individual neurons of the nerve substance, and a striking analogy between them in their relation to electric energy and shock, which may throw light not only on the physiology, but on the cause and cure of the functional neuroses. It is well known that we can hasten the recovery of hysterical paralysis by placing the patients in an electro-static field, or subjecting them to the influence of currents of high potential and high frequency and by other forms of electric energy. The analogy between the two—the tube of iron-filings and the nerve structure—may be carried still further, since the restored nervous conductibility in a case of paralysis of hysterical origin may be instantly annihilated, as is the electric conductibility in the tube of iron-filings by sudden shocks. "Does not this help to make clear to us the characteristic causation of hysterical paralysis? Does it not reveal the traumatism which produces them? Moral or physical traumatism, emotional shock or railway accident, psychical influence or injury, brutal application or physical agents, heat, cold, light, etc., in fact, all violent excitation is capable of producing less of conductibility of the nerve current proper, in or through the neurons, as it can restore it when abolished." In certain cases of hysterical paralysis patients who have been confined to bed for months or years have suddenly regained power when some violent neuro-motor excitation, such, for example, as a conflagration, has overcome the non-conductibility of the neuron. Whether or no, this discovery is sufficient to furnish the key, as its enthusiastic advocates suggest, to the cause and cure of the functional neuroses, it at least opens up a novel and interesting field of research.

---

***NOTICE.—This JOURNAL and THE INTERNATIONAL JOURNAL OF SURGERY one year for $1.00 cash, to new subscribers. Out-of-town checks not accepted unless 10c. is added for cost of collection. Strictly to new subscribers.***

### THE MODERN TREATMENT OF INCIPIENT PULMONARY TUBERCULOSIS.*

I have taken as the subject-matter of my paper the treatment of the preliminary stages of pulmonary tuberculosis, and I propose to limit myself to this phase of the disease. We are confronted in discussing the nature of tuberculosis with a devastating affliction of the most deadly, the most virulent, and the most wide-spread of all diseases. It has been estimated that one-seventh of the mortality of the whole world is due to it alone, and the insidious nature of tuberculosis renders it not only difficult to handle, but presents obstacles in the way of its early detection. It kills not by a long-continued systematic poisoning, but by pathological changes, brought about through the localization and growth of the germs in organs necessary to life. To Robert Koch, of Berlin, is the credit due of having isolated the tubercle bacillus. It will be my duty to enter, with some detail, into a description of the features of this disease, and some of the essentials of treating it. Within the last few years our knowledge of the functions of the ductless glands has become much enriched, and we now ascribe to them physiological influences of immense importance. We are but on the threshold of an understanding of their objects and value, but, as in the case of myxedema, the history of which we may be now considered to be fairly well familiar with, there can be little doubt that further research will reveal that each ductless gland fulfills some useful duty in our economy. With this view of the value of the ductless gland in mind, I never could persuade myself that in the treatment of enlarged tonsils a complete excision was altogether wise. I felt that the tonsils fulfill a mission, one part of which was to act as a first line of defense, and, like a military outpost, to resist attacks from hostile forces. Metchnikoff's demonstrations of phagocytosis lends itself to this view. Whatever other functions these glands may have are at present too hypothetical to be discussed. To my mind each gland represents a fort in which leucocytes are housed, ever watchful to exert a defensive resistance on parasitic micro-organisms. In such a

* Read by Louis Henry, M.D., Melbourne, before the Victoria Branch of the British Medical Association, August 15, 1900.

disease as the one under consideration, the leucocyte has an important rôle to perform, he not only defends but he attacks, for he has the power of taking into his interior living bacteria and destroying them. In conjunction with this cellular method of defense, which is both phagocytic and chemical, there are other influences at work which are gradually losing their purely speculative character. I refer to the recuperation of the nerve cell after exhaustion and to other evidences of the active implication of the tissues. In the struggle that ensues, sometimes the virulent bacillus wins, and sometimes the leucocyte is victorious, a matter of deep concern to the host; but what the qualities must be to assure success to the leucocyte and prevent the other agencies of protection of the individual from being overtaxed, is one of the objects which this paper sets itself to explain.

In olden times, to navigate a vessel on a long ocean cruise, when instruments of precision were unknown, was an undertaking of some magnitude. Those mariners who traversed the seas carried with them only their astrolabus, which they used for taking the position of the sun. Observations of the heavenly bodies served them to find the longitude, while a dead reckoning was the only means by which their latitude was ascertainable. But for all this, those ancient sea-fighters, after struggling with unknown currents flowing in devious directions, were not so much out of their course. Their method of navigation was slow, not to say risky, and we cannot withhold our admiration to those pioneers on the sea whose keenness of observation, and whose graphic powers of accurate description have left us but little to add to. Nowadays, with our chronometers, our sextants and quadrants, with our knowledge of ocean currents, with all the armamentarium at our disposal, we certainly have reaped a large field of research, gathering abundant harvests and enabling us to bring the most distant countries into touch and unite mankind in one brotherhood. And so the physician of to-day should not be ungrateful to those pioneers of medical science who, reliant upon their own independent research, and frequently without co-operation, have contributed so largely in establishing the foundation of that great storehouse of knowledge from which we gladly borrow the fundamental principles of our art. In no affection will the broad principles of treatment based upon sound pathology stand out more prominently, and in no instance in the realm of medical therapeutics will the value of intelligent guidance on the part of the physician be more conspicuous than in the handling of tuberculosis. The utility of narrow limits of ideas by a treatment of antiseptics or specific remedies may, for a time, be apparently successful in treating special symptoms, but they are after all only palliative, and the results, it will be found, are only purchased at a heavy cost. Something must be sacrificed at the expense of temporary relief. The food intake will be less, the appetite becomes diminished, nutrition must suffer, and one of the chief agencies of treatment is constantly hampered. The great ideal to be arrived at in treatment, it will be seen, is to invigorate the patient's power of successful resistance, to stimulate the vitality of the individual to rapid assimilation and repair, and to keep the mucous surfaces normal. These objects, in conjunction with oxygenation of the blood, a good metabolism, a natural excretion, are the principles which must direct treatment in arresting disease and promoting general health. We must discard the idea of the disease being the entity; it is the patient who must be paramount.

In a paper read before this branch in 1892 Mr. Candler observed that an effective system for prevention of consumption is not to be devised without the full knowledge of its causation. Koch's historical address was delivered in 1882, at Berlin. In every step connected with the discovery of the tubercle bacillus, and with the experimental proof that the species is the infective agent in tuberculosis, is seen the work of the most consummate micropathologist of the day. The parasitic nature of the tubercular virus having been accepted, some inquiries may be made :—Firstly, where do the parasites originate? Secondly, how do they gain an entrance into the body? As the result of his experiments Koch declared that the tubercle bacillus could grow only between the temperatures of 86° F. and 105.8°. Under 86° F., and at 107.6°, no growth took place during three weeks' time. It follows that the tubercle bacillus, in its process of development, is limited to the animal body; moreover, it is not an accident, but a true parasite, that can originate only in an animal organism. How do the parasites enter the body? The majority of all cases of tuberculosis have their beginning in the respiratory tract, and the infectious material becomes apparent in the lung or bronchial gland. It is therefore very probable that the bacilli are inhaled into the lungs by particles of dust. The manner and way in which the bacilli become mixed with the air is not so very mysterious, particularly when we consider the quantity of bacilli in pulmonary cavities, which, mixed with other organisms, are expectorated and disseminated all round. Sputum, after drying, does not lose any of its virulence, and entering the lungs as dust it is the cause of tuberculosis.

Koch states that the tubercular organism finds conditions suitable for its existence only in the animal body, and that it cannot exist under ordinary conditions outside of it. As a prophylaxis he lays great stress on the recommendation that those sources from which the material of infection come should be closed, the chief of which is the sputum.

In 1887, and again in 1892, Mr. Candler published his ideas on what he considered a radical error in Koch's view of phthisis. He says, "that because Koch did not succeed in getting the bacilli to grow below 86° F. it was absurd for him to suppose that no other micropathologist would succeed." Mr. Candler believed it quite possible for the bacillus to grow outside the animal organism, and he discarded the idea of the purely parasitic character of tuberculosis. It is his opinion that this specific vegetation occurs naturally in the flora of most habitable regions of the globe, and is carried by means of the air into the dwellings of man and animal. It then establishes itself on suitable soil, and it is by means of breathing air containing the seeds of the growing saprophytic vegetation, and not by inhaling an atmosphere holding the diseased spores of the parasitic stage of the species, that phthisis is contracted.

Although Koch believed that the tubercle bacilli would grow only in media containing animal fluids outside the animal body, not below 86° F., it was shown by Pawlowsky that growth will take place also on a purely vegetable medium. Sander discovered that growth took place in potato broth even at 71.6° F. and 73.4° F. While it seems doubtful whether nature can supply all the necessary conditions for the growth of the bacilli, there is every evidence to show that their great culture-ground is the animal body, and the agencies by which tuberculosis is spread are the secretions.

The power of resistance of the tubercle bacilli is very considerable, and outside the body they are quite able to sustain their vitality for a very long period. The action of direct sunlight is rapidly fatal to them, and they are able, in the dried condition, to resist a fairly high temperature.

The chief methods of infection are by inhalation and ingestion of food contaminated with tubercle material.

The whole of the field of bacteriological inquiry leaves still room for further research, but this much has been learnt that pulmonary tuberculosis is a mixed infection. It is quite possible to become confused with the innumerable classifications of this disease, for one is in great danger of losing sight of that unity of principle with diversity of result, which underlies and explains the varying aspects of phthisical and chronic diseases of the lungs. One of the best definitions of consumption, although it practically excludes miliary tuberculosis, is that of Sir Andrew Clarke; it is as follows: "An assemblage and progression of symptoms associated with, and dependent upon the ulcerative or suppurative destruction of a more or less circumscribed, non-malignant deposit in the lungs." I desire to quote another definition, possibly more comprehensive: "The tubercular bacilli excite at the place of localization the formation of small granulations, tumors, tubercles." These consist of an accumulation of leucocytes and hyperplastic fixed cells of the tissues. These tubercle nodules undergo necrosis (caseation) and disintegration, leaving ulcerous areas that extend and become confluent, so that the affected organ is gradually destroyed. Frequently the tuberculous ulcer becomes the seat of secondary infection (mixed infection), with pyogenic cocci and other micro-organisms, which in turn bring about secondary inflammatory processes (infiltrations, septic diseases). The cure of the tuberculous process takes place through absorption, connective-tissue hyperplasia (cicatrization), and calcification.

As a rule, tuberculosis is a localized infection, and may begin by infecting the pharyngeal tissues, tonsils, the lympathic glands of the neck, lungs, intestinal tract, bones or skin. The infection of the larynx is usually secondary to that of the lung, and very often there exist cryptogenetic foci deep down in the parenchyma of some organ such as the intestinal glands, fallopian tubes, etc., where the bacilli may remain latent for a longer or shorter period. The most frequent form of infection, however, is in the lung, usually in the upper part, or more frequently in the left apex. Acute miliary tuberculosis is a generalized condition where various viscera are implicated, due probably to the rupture of some localized focus containing large quantities of living bacilli, which, pouring into the blood stream, spread in various directions throughout the system. Individuality and a predisposition to tuberculosis are admitted to exist. This predisposition may consist in some constitutional weakness, in a tendency of the tissues to catarrhal troubles, in some lesion of the mucous surfaces, in some mal-development of the thorax or in some other obscure cause. It is not at all unusual for the tubercular process to remain local. This may be due to arrest of growth and possibly some restraint in development, owing to a healthy resistance on the part of the invaded tissues. The presence of tubercle bacilli in milk is detected by centrifugalizing the milk and then by examining the deposit microscopically or by inoculating an animal with the deposit. It is this infected milk which is the greatest source of tabes mesenterica in children. Swallowing sputa will give rise to intestinal infection in adults.

A difference of opinion still exists among many as to the unity or duality of the origin of consumption. Niemeyer is the exponent of the dual theory. Laënnec is the champion of the monistic cause, and he states that pulmonary phthisis is a constitutional disease, and that it never can develop itself out of acute or chronic pneumonia, or take its rise from a bronchial hemorrhage, or from a neglected or protracted cold. Niemeyer teaches that consumption is not, in all instances, due to a specific growth, but may arise in two different ways. According to him, consumption and phthisis are synonymous terms which describe the lung affection. He restricts the term tuberculosis to a much narrower field, allowing its application to only that form of phthisis which includes a deposit in the lung of the specific new growth which he calls miliary tubercle. Inflammation of the lungs, and its termination in cheesy metamorphosis is the one cause, and the development of the tubercle (miliary tubercle) is the other. He declares that the large cheesy masses, which break down and form cavities, are not tubercular, but result from purely inflammatory processes. He holds that consumption is more often due to chronic catarrhal pneumonia than to any other one cause. The second form is that in which "tuberculosis has associated itself with the phthisis." Tubercles, he goes on to say, are generally a secondary product, and tuberculosis is mostly secondary to the action of cheesy, morbid products. He further dogmatizes as follows: "The greatest danger to most phthisical patients is the development of tubercles." Primary tuberculosis, according to him, is a new growth. After insisting upon the curability of many cases of consumption, he says, "Against that form of phthisis, which consists in a primary tuberculosis, as well as against the tuberculosis which has been developed in the course of phthisis, treatment is indeed impotent." Although Niemeyer's contributions towards medical science have received every acknowledgment and must not be underestimated, still, examined by the light of modern investigation, his train of argument will not hold, and his conclusions cannot altogether be accepted. Koch's discovery renders it almost impossible to believe in the duality theory. At present Koch's dictum is to the effect that consumption of the lungs is the result of a specific new growth, which is caused by the bacillus tuberculosis.

Opponents to this doctrine declare that it is inadequate to explain all the phenomena of tuberculosis. Sir Andrew Clarke makes the following remarks: "It has been alleged by Koch—and is generally believed in London—that every case of phthisis is microbic, and is associated with, and dependent upon the presence and the action of tubercle bacilli. I presume to deny the allegation, and to contend that whilst the great majority of cases of phthisis are bacillary, there is a considerable minority of cases which are non-bacillary, in which at no period of their history can bacilli be found." Many reliable clinicians assert the same. Dr. Meigs expresses it as his opinion "that it is impossible to escape the conviction that there are cases of consumption, especially some of the more chronic forms, and usually classed as fibroid phthisis, that cannot possibly be due to either the bacillus or to any other infectious cause. These cases must arise from some disordered action of the bodily organism itself, or, to be more precise, that they result from inflammatory action—inflammation being given a broad definition, making it cover that which occurs in the tissues after a great variety of forms of injury—a result of ill-ordered growth and disintegration of the natural component parts of the organism."

The prognosis admits of a more hopeful view than was formerly possible. The treatment now followed is based on a precise pathology. Our chief aim, as it is now in many other diseases, is to increase the tissue resistance of the individual—to improve his vitality—to place him in an environment which will both help to neutralize the attack and to raise the patient's mental and physical tone. Alcohol habits increase a susceptibility to contract phthisis. Pregnancy retards changes in the lung, while lactation aggravates it, and so quickly may phthisis come after parturition that many deaths from consumption are put down to childbirth. Diarrhœa, as a consequence of tuberculous enteritis, and laryngeal tuberculosis, are both grave symptoms. Edema and thrombosis of the veins of the legs and aphthous stomatitis portend the end. In this disease, as in many others, debility and diminished tissue-resisting powers of the body permit of fresh invasion from both within and without, and so induce composite symptoms of a fresh series of apparently independent diseases.

Actual hereditary infection is brought about by the transmission of the bacillus from parent to embryo. The infection may occur by the passage of the bacillus through the placenta; by the infection of the ovum from the internal tissues or fluids; by infection carried in the fructifying sperms. By whichever means the infection is brought about, the activity of the bacillus may remain latent in the individual till some cause provokes it out of its quiescent stage. Intra-uterine infection, however, is rare, but its possibility is borne out by careful investigations, more particularly in experiments on animals. Much of what at one time was thought to be inherited phthisis is to be regarded as not in any way due to direct transmission, but is the result of effects of the same environment on the individual, and proceeding from direct infection by the inhalation of phthisical dust and from the general contamination unavoidably associated with living in an infected atmosphere. The offspring in this way acquires the disease, as his own resisting power becomes weakened, whereas the same individual, if removed timely to healthy surroundings, and away from transmitting centers of infection, would escape altogether. Parents should be taught that it is not by inheritance, but by infection, that the disease is spread, and they should be urged to take timely precautions to save the family.

The earlier symptoms of pulmonary consumption are very often attended with difficulties of diagnosis. Long before physical signs in the lung become sufficiently accentuated, incipient disease may exist. There may be no expectoration for bacteriological tests, and, in fact, no objective condition to help one. The temperature may be normal, and nothing but subjective complaints be heard of. Very suggestive of the approach of disease are evidences of dyspepsia, slight hoarseness, loss of weight, a sense of weariness, a want of recuperative power, and, in fact, a general feeling of malaise. Cough is not an early symptom, but diarrhea may be. Acute pleuritic effusion is always suspicious, and very often anemia may be the only clinical sign. At a very early period auscultation may distinguish prolonged expiration due to a loss of elasticity of the lung. The alteration of the percussion note follows, and fine moist râles may now be heard. The sub-clavicular dulness, the amphoric breathing, etc., are all symptoms of later stages. In the primary stage dulness is not obtainable for the small tubercles, and the circumscribed pneumonic patches affect only separate lobules or alveoli, so that the percussion note of the chest is barely affected. When, however, lobules lying together become densified, it is possible to recognize a dulness in tone. A large cavity at the apex may be a cause of dulness, as these cavities are frequently surrounded by fibrous tissue, which may be adherent to the chest wall; these cases offer a favorable prognosis. When, however, the dulness is less marked, and is accompanied by prolonged expiration, râles, fever, and wasting, the prognosis is bad.

A recent case under my own observation presented itself with all the difficulties of obscure origin, and with almost complete absence of all specific physical signs. X. Z., aged forty-six, had rigors, neuritis of the scalp, dyspepsia with vomiting, and pains in ankles and knees. Pulmonary symptoms were not detectable; there was no deviation from the normal temperature until a fortnight after the first rigor. The first indication of pyrexia was an evening temperature of 100°, and from that period for fully ten weeks the temperature fluctuated up and down—the morning temperature between normal and 100°, and evening rises between 101° and 103° F. The pulse itself, in the horizontal position, varied between 70 and 100, and beyond the pains already mentioned no other manifestations were evident. On account of the total absence of both subjective and objective pulmonary symptoms the diagnosis was presumed as being influenza of a rheumatic and typhoidal character; one reason for favoring influenza was the protean type of the attack. Auscultation with the binaural stethoscope detected a difference in the respiratory sound of the two lungs. From the very first day of my examination of the patient I was suspicious of the respiratory murmur of the middle lobe of the right lung, but no development of this condition was manifest. There was no cough, no pain in the side and beyond the pyrexia, rheumatic pains and coated tongue there was nothing to show. The resonance of the lung was not diminished, nor was fremitus increased. It was only at the end of eight weeks, after frequent examinations of the blood for typhoid by Widal's test, which were negative, that my first impression of implication of the lung was corroborated. About this time the patient showed another objective symptom, which, however, in itself could not be taken as pathognomonic, namely, day and night sweats with slight wasting. There was nothing for bacteriological examination, there was no other indication of possible tubercular infection. Taking, however, the whole character of the case, I was encouraged to favor the idea of it being tubercular, and although the night temperature still occasionally went over the 100°, I ordered his removal away from home to a more favorable climate, and gave instructions for the carrying-out, in a modified form, of the open-air treatment. Long before this I had begun to feed the patient up with nutritious food. The result of this treatment has been eminently satisfactory. He is increasing rapidly in weight, he is able to take outdoor exercise, to do a little shooting, and for days at a time his temperature remains quite normal. Exacerbations occur occasionally, when he presumes too much on his strength and indulges in overexertion. Other cases which I have had have given me every encouragement to persevere in recommending a similar mode of treatment.

One of the most powerful means of combating the tubercle bacilli is fresh air. The patient must be rescued from all elements of depression, from atmospheres permeated with the products of respiration, and from dust, and he must be continuously exposed to pure air both during day and night. The temperature of the air itself is not of such importance as its purity, neither is barometrical pressure anything but of secondary importance. Brightness and sunshine are factors to be sought so that outdoor life may be conveniently and comfortably entertained. Cough and expectoration should not be checked altogether, and while neither should be unduly controlled, still, if distressing and harassing, must be relieved. Exertion and fatigue are to be watched, as both are liable to induce aggravation of temperature. Fever must be treated by rational methods, and powerful antipyretic drugs are to be avoided as useless and lowering. All debilitating causes are to be investigated and attended to. The condition of the stomach must be enquired into, and, hand in hand with the importance of free air, a form of nourishment, suitable to the patient, is to be advised. In this manner, where the disease is of a limited extent, it may be arrested, early aggravations are checked and the patient is placed in the most favorable circumstances for his recovery. There is no chapter in the whole realm of medicine where the medical attendant is called upon to exercise a keener judgment, founded on accurate observation, and where, realizing the full responsibility of his duties, he is better able to take advantage of a rich experience and obtain a gratifying result. To sum up, attention should be directed to digestion, to food, to free oxygenation of the blood by means of fresh air. The normal resistance of the tissues is to be regained, catarrh and local congestion are to be thrown off, and the soundness of the mucous surfaces preserved. Pathologically speaking, the treatment consists in the observation of strict hygienic influences which have the effect of inhibiting active destructive processes in the lung, such as subacute consolidation, softening, caseation, and ulceration. That such effects in arresting the disease are produced is known to all those in the habit of making post-mortem examinations.

Nature shows its method of reparation by throwing out a fibrous material as a first process of regeneration. A natural protection is thus afforded, cicatrization is encouraged, necrosis is checked, and ulcerations heal. The further effect of fibrosis is the encapsuling of the foci and the culture beds of the tubercle bacilli, so that they practically die in their own toxins. Sometimes it happens that the diseased material becomes converted into a calcified and calcareous mass. At any time, however, owing to lowered health or accident, an enclosure may rupture, then a fresh invasion takes place. We must not overlook the fact that fibrosis may outrun its usefulness by limiting the respiratory power by its interference with the elasticity of the alveoli. Fibrosis is therefore not an unmixed good and its activity requires watching.

The principle of the treatment adopted by Dr. Walther, at the Nordrach Sanatorium, is as follows:—Hemoptysis is regarded as an accident and treated as such. Fever is not considered as a contraindication for food, and improvement of nutrition is considered as a key to the situation. Each case is treated according to its individuality. Food is directed to be given as much as possible, exercise is ordered in a systematic manner, as it improves condition and throws into activity the healthy part of the lungs, and so the circulation and respiration is encouraged, and as occupations of a cheerful nature are indulged in, brooding is avoided. When pursuing this treatment, clothing should be worn of a warm and light texture, and good flannel next to the skin is preferable to any other material.

The following is the regimen:—

Breakfast at 8 a. m.—Tea or coffee, cold tongue, ham, fowl or sausage, bread and butter (with plenty of the latter), and one pint of milk.

Dinner at 1 p. m.—First course: fish, fowl, or meat. Second course: Fowl or meat. With both courses a liberal supply of potatoes, vegetables, and gravy or sauce, with butter as a main ingredient. Third course: Fruit with biscuits, nuts, etc.,—say three times weekly. The other days pastry, milk puddings, ices, etc., with coffee and one pint of milk.

Supper at 7 p. m.—One hot course as at dinner, potatoes and vegetables included, and one cold course as at breakfast, with bread, butter and tea, with one pint of milk.

The patient must weigh every week. It is advisable for him: (1) to eat as much as possible at meals and nothing between while; (2) to have a long interval between meals, so as to allow a complete assimilation of food; (3) to take one hour's rest, reclining on a sofa or in a hammock, before dinner and supper. Smoking is allowed in the open air.

Regulation of Exertion and Rest.—The patient must be entirely guided by the condition of his temperature in this matter. Temperature to be taken on each occasion in the rectum, preferably on waking in the morning, then after the morning walk (or if resting at 11:30), again after the afternoon walk (or if resting at 5:30), and at night ten minutes after retiring to bed, which should take place at 9 or 9:30. The temperature is to be taken after walking, when exertion ceases the temperature usually drops. If temperature is not over 98.6° in the morning, and is below 100.4° in the evening, after rest, a gentle walk may be taken. If, however, it shows above 98.6° directly after waking up, and if it reaches 100.4°, or even 100° in the afternoon after rest, it is too high, and the patient must have complete rest on the sofa each day until the temperature comes down. When the patient is at rest, and the temperature reaches over 100.4° in the evening, it indicates that he needs absolute rest in bed and alone in his room. Under these circumstances even talking is to be avoided. The same quantity of food is to be taken when in bed with fever as when about. The more food taken the sooner will the fever abate. If the morning temperature keeps regularly below 98.6° a short walk (say half a mile), at a uniformly very slow pace, may be taken before breakfast. If after this exercise the temperature rises above 100.4°, then the walk has been too long, and rest must be taken on the sofa for the remainder of the day. Patients may read, but not fatigue themselves in any way. The next day a shorter walk is ventured. Then, when the temperature after the walk is well below 100.4°, and if no fatigue is felt, a short stroll may be taken in the afternoon after dinner. The repetition of this is governed by the temperature obtained on returning from the walk. As strength increases the length of the morning walk may be extended. As much time as possible should be spent in the open air. Rain, sleet or snow should not keep the patient indoors. If caught in a shower he should not hurry, breathlessness produced by hurrying is bad. The windows of the rooms and sleeping apartments must always be kept as full open as they will go, day and night, summer or winter, in every kind of weather. The patient need have no fear of catching cold if he will always live in such rooms and avoid those which are heated and close. Cold is caught by infection, not from draughts or wet clothes, which more than the disease cause the coughing. Cough will soon leave when natural open-air life has begun. When resting the patient should sit close to the open window, or in the garden. If the air is cold he should have a rug wrapped round his feet and legs. When walking he should have as little clothing on as possible; he must gradually lay aside all chest-protectors, double flannels, overcoats, etc. The less weight he has to carry the better. He should have ten hours' sleep each night, and must sleep with bedroom windows wide open. If cold he may put more clothing on his bed. Every patient should have a bedroom to himself. He must avoid heated rooms—concert halls, theaters and churches. He must give up dumb-bell and all such suicidal exercises. The bedrooms are well ventilated, and a good draught passes through the bedrooms during the night. In erecting a sanatorium, in selecting a site you may go to the highlands, or the lowlands, or the bogs; it is of little consequence where you go. It is no use coquetting with climate; the climate has nothing to do with the matter. All that is absolutely necessary is: (1) A spot in the country where pure air is to be had; (2) well away from smoke, dust, traffic, and excitement, where the patient may lead the quiet unconventional life so necessary to his well-being; (3) the proper treatment to be conscientiously carried out; and (4) the most important of all, the patient to honestly cooporate in attending to all details; (5) the principal outlay of money should be on the patient, and not on the buildings. It matters little whether the sanatorium is at sea-level or on a mountain, whether there be little or no wind, whether the walks be up hill or down hill, only fatigue must be guarded against. It matters not if there be much or little sunshine, whether the rainfall be high or low, whether the soil is porous or otherwise. All places in the country where pure air is to be had are equally healthful. The ideal place in the country is five or six miles from the nearest village, about 500 feet to 1,000 feet high, protected from the sharp bleak winds by hills, and open to milder currents of air, with plenty of trees, which will afford protection from wind and sun, where good expanse of country is to be had, where the patient may roam freely about. There must be no stinting in the matter of food.

One of the great difficulties of the open-air treatment is the length of time during which it must be carried out. I consider the sanatorium should be erected at an elevation of some hundreds of feet, and away from any town or factory, with a protection from the coldest winds on a good well-drained soil. Sunlight must be free and accessible, and there should be facilities for walking among trees and sheltered groves; the incline of the paths, if any, should be from the sanatorium. The food should be plentiful, nutritious, and well cooked. The lighting in the household should be by electricity, and a uniform temperature obtained by means of hot-water pipes. Ventilation should be good, and there should be a free admission of sunlight into the house. Everything that harbors dust must be avoided, and facilities for rest must be afforded.

The cows supplying milk should be regularly tested at least every six months. Medicinal treatment should not be excluded altogether, and the value of such therapeutic weapons as iodine, morphia, iodide of potassium, must not be forgotten.

The value of sanatoria is being demonstrated every day. Many of those who have been treated there, providing their stay has been sufficiently prolonged, maintain for many years good health, and are able to resume work.

The value of the treatment at a sanatorium cannot be overestimated, as a visit to a special institute inspires faith and hope in the patient; the patient quickly acclimatizes himself to his new surroundings. Temperature is gradually reduced, the appetite is increased, night sweats disappear, and regular exercise may be taken. I do not claim for the open-air treatment that it will cure all cases, but, without doubt, in those early stages of disease with which we are at present dealing, a much larger percentage is benefited than by any other treatment.

In conclusion, I would like to point out the urgency that exists for making some provision for that large class of patients who suffer unknowingly from those earlier stages of consumption, and who seem still well enough to follow their occupations. In the course of events, they are bound to drift into a less amenable state for treatment. As productive members of the community, and as bread-winners to their families, they stand, as things now are, very little chance of recovering their usefulness. Their apparently slight ailments which they at first struggle against, appear to them to have neither the significance nor the gravity which we know them to possess. The congested out-patient departments of our hospitals offer them no relief, nor can sufficient time in most instances be given to these cases for correct diagnosis to be arrived at and advice to be tendered. Within reasonable distance of every center of population some public sanatorium should be established, where the attention can be supplied which these cases have a right to demand. In this way only is it possible to save valuable lives for the community, and to apply treatment to those poor unfortunates whose inevitable doom must otherwise lead them on to an incurable stage of their complaint.

The treatment I have attempted to describe comes really under the heading of what might be termed "hygienic" treatment, for its rationale practically depends on the effort of strengthening the organism to effect its own cure. The attempt of destroying the bacilli and neutralizing their products from without, by the administration of chemical germicides, antipyretics, serums, and antitoxins, has been superseded in favor of the living tissues accomplishing this work successfully from within. The individual, who surrenders himself to skilled medical supervision, has every reason to be buoyed up, when he can have the assurance made to him that "Nature" has endowed him with all the essentials requisite to produce a curative result.—*Australasian Med. Gazette.*

---

### SOME EXPERIENCES IN THE WAR IN SOUTH AFRICA.

A great war necessarily presents many phases and points of view, and as the time allowed for the reading of papers by associations, such as I have the honor to address, is limited, I shall only deal briefly with one or two phases which I hope may prove of interest.

The war in South Africa is interesting surgically because of the experience which has been gained of the effect of modern arms of precision, and of antiseptic methods on the field of battle and in the hospitals. It is too early yet to draw deductions from the statistics of the war, but it may be noted in passing that while 936 officers and 11,701 non-commissioned officers and men have been wounded—12,637 in all—only 732 have died of wounds received in action, an infinitesimal proportion, which may be fairly ascribed to the aseptic character of the bullet, to the prompt application of a first aid dressing and to the able and eminently efficient treatment which the wounded received at the hands of the medical officers in the hospitals. The Mauser bullet has justly been described as a merciful one. Its action upon human tissues depends, however, upon the range at which it is fired. It has been noticed that when it is fired at short ranges, within two hundred yards, it has an explosive character. The nickel case seems to expand and become detached, causing a severe lacerated and contused wound, which heals but slowly. If it strike bone it crushes and destroys it. If fired at longer ranges it makes a clean drilled hole in bone, and if it strike soft parts only a very small wound is made, there being little difference between the wound of entrance and that of exit, which bleeds but little unless an important vessel is injured. In the case of the soft-nose or dum-dum bullet the wound is much more severe, for even where the soft parts only are injured the expansion of the lead causes great destruction of parts and a huge wound of exit, the wound of entrance being small. When it strikes bone it pulverizes and disintegrates it. If the range is very long, 2,000 yards or more, the soft-nose bullet "mushrooms" and causes an extensive flesh wound. It has been alleged that poisoned bullets were used. I have seen many of these so-called poisoned bullets. They are simply green with verdigris, which in all probability is burned off in the rifle while the bullet is in transit through the barrel. I have heard of no case where poisoning by a bullet could fairly be said to have occurred. It has been charged that explosive bullets have been used. I very much doubt the fact. The explosive character of Mausers at certain ranges has already been referred to. It is probably this which gave rise to the statement. When a Mauser bullet strikes a hard substance at a short range the impact is terrific and causes the bullet to fly into a thousand pieces. Besides Mauser rifles the Boers made use of many thousands of Martini-Henrys. As is well known, the bullet is a heavy one, and where wounds are inflicted they are in striking contrast to those inflicted by the Mauser. Great destruction of soft parts or bone follows, necessitating amputation in many cases. It is remarkable how few amputations have been performed during this war. Dr. Kendal Franks told the writer that in his experience not more than twenty amputations had been done in 3,000 cases, which must be attributed to the character of the wounds and to the conservative spirit of the surgery of the day. I had the opportunity of examining a good many Boer wounded and found that the bullet of the Lee-Metford rifle inflicted a wound very similar in character to that of the Mauser. It is not necessary, therefore, in this place to say anything more on that head. The effect of shell-fire was interesting, if not destructive. The Boers say it is no good and only makes one keep one's head down. The lydite shells are not nearly so destructive as was supposed. When they strike soft ground they do not explode. When they strike a rock they explode with great violence, but our friends the enemy were so cleverly entrenched that but few were injured by them. I remember seeing one man stained a bright yellow from head to foot and apparently not much the worse for it. They said the escaping gas made their heads ache and they found that a few drops of vinegar taken inwardly relieved it. Every Boer was provided with a small bottle of vinegar to ward off the ill effects of lydite. I have said that I had the opportunity of seeing a good many Boer wounded, and as their condition presented special features of interest, I will venture to detain you a few minutes by referring to some of the cases I saw. When I was at Kimberley we had 147 Boer wounded in the roller rink, which had been converted into a temporary hospital. They were of all ages, from fifteen to sixty-five, and bore their captivity and sufferings with dignity and patience. They had been wounded at Paardeberg chiefly, and in many cases the wounds had been undressed for sixteen or seventeen days. I remember one man who had been shot through the elbow joint. His only treatment had been the universal Boer remedy, tobacco juice. The arm was enormously swollen and almost erysipelatous in appearance. Mr. Roberts, civil surgeon, opened up the joint freely and removed considerable masses of bone, and found a large piece of shell so firmly imbedded in the humerus that it could not be removed. The wound was very offensive. It was freely drained and douched with bichloride, dressed antiseptically and supported by a rectangular splint. This man made an excellent recovery, with a movable joint. Another man was shot through the body by a round ball from a shrapnel-shell. The projectile entered four inches below and to the left of the heart and came out through the sixth intercostal space on the right side and posteriorly. He had been wounded sixteen days previously, and at the time that I saw him he had practically no symptoms. This is the more remarkable when the character of the missile is considered. I have seen a good many penetrating wounds of the abdomen which have produced little immediate disturbance. One was the case of a medical officer who was shot through the stomach. He had little to eat for twelve hours before his wound. When he was wounded he had the sense to abstain from drinking, notwithstanding the urgent thirst; and further, he lay still where he was for twelve hours. He made an excellent recovery, but I observed that some three months after his wound had healed that he complained of dragging and discomfort in the neighborhood of the wound, and was eventually invalided home. Two of his companions who had been shot through the abdomen at the same time were so unwise as to drink water and died in a few days from peritonitis. This may have been caused by the filthy water. It was generally understood in South Africa that abdominal section in wounds of the intestine was inadvisable, judged by the results; but I know of one case where an excellent result was obtained. It was surprising how great joints like the knee could be pierced by Mauser bullets with impunity. I remember the case of an officer who was shot through the knee-joint at the battle of Korn Spruit. In six weeks he was walking about without a crutch, and had returned to duty. Ten days later this officer, with his squadron, was ordered to take a kopje. His brother officers were killed beside him, and he was shot again in the same knee. The wound proved to have been caused by a spent bullet, and was unimportant, though the missile had lodged in the patella tendon. Wounds of the chest by Mauser bullets were comparatively innocuous, and healed readily. In some cases there was a considerable hemorrhage into the lungs, with marked difficulty of breathing. If the obstruction was not so great as to seriously interfere with respiration, these cases recovered. Wounds of the head were necessarily more serious, but I observed several in which there was considerale destruction of brain tissue, which afterwards made good recoveries. One case was that of a boy who was shot in the left side of the skull, close to the coronal suture, the wound running anteroposteriorly, and who had right hemiplegia, from which, after removal of fragments and depressed bone, a good recovery was made. A young Boer was shot through the mastoid, the ball coming out just above the zygoma. He made an excellent recovery, with impaired hearing. I notice that many apparently minor cases of grooving of the outer table of the skull were accompanied by reflex symptoms. Wounds of the eye were of frequent occurrence, and in most cases were most destructive. I saw two cases in which both eyes were lost, the bullet passing through the orbit and cutting the optic nerves. Another man had the optic nerve cut on one side, and, strange to relate, on the nerves of motion, cut on the other.

There was almost complete ophthalmoplegia and mydriasis, but the vision was otherwise unimpaired. Cases of injury from fragments of shell and sand, thrown into the eye by exploding projectiles, were very common. Altogether, wounds showed a surprising tendency to heal rapidly, even under the most trying circumstances, which was due to the character of the bullet, the early application of an antiseptic dressing, enforced temperance among the troops, their general good health, and the careful and painstaking work of the Royal Army Medical Corps.

Before closing I wish to make a very few brief remarks upon the hospital administration in South Africa—a subject which is occupying a large place in the public mind because of the attacks which have been made upon the administration under the guise of philanthropic interest in the welfare of our soldiers, but which is in reality a thinly disguised and discreditable political attack upon the Imperial Government. The position was this: The Orange Free State is a land which produces little towards its own support in food for men and horses. Its main artery of communication is a narrow-gage railway, of the length of 725 miles, between Capetown and Bloemfontein, where the alleged atrocities are said to have taken place. Suddenly a hundred thousand soldiers and twenty thousand camp-followers are thrown into this country, already bare and hardly able to feed itself. Add to this twenty-five thousand horses, mules and oxen, all of whom, men and animals, have to be fed by this narrow-gage railway. In addition there are munitions of war—horses, mules, guns and soldiers to be carried, besides miscellaneous hospital and personal stores, passengers, as well as food and merchandise for the residents of the country. With a limited rolling-stock I leave it to you to imagine how difficult was the problem which confronted our army; a problem which was rendered still more difficult by interruption of communication by the blowing up of bridges. Then, almost without warning, a great epidemic of enteric fever broke out. In one day upwards of a thousand men were admitted to the hospital.

Would it be surprising that beds and bedding were hard to find, or that orderlies and nurses were overworked? Naturally, under the circumstances, the field hospitals had to be utilized as stationary hospitals, though they are neither equipped nor intended for such work. The officers and men of the Royal Army Medical Corps rose to the occasion, and did magnificent work, heroically sacrificing themselves on the altar of duty, as is proved by the death and disability returns. In short, everything was done to meet the requirements of the emergency that circumstances permitted of, and there did not exist the neglect and misery so graphically and glibly set forth by certain untrustworthy politicians. No one was more keenly interested and sympathetic than the Commander-in-Chief, Lord Roberts, and I always found him most willing to grant every reasonable facility in getting up stores and comforts, and in aiding our work in every way.

The history of this war redounds to the credit of the medical officers, civil and military, who worked so faithfully, so energetically, and so successfully to alleviate suffering and assuage pain. The medical organization of the army is by no means perfect, and will require readjustment when the war is over. The medical officers should be given entire control of their supplies of medicines and drugs. They are now supplied by an ordnance department. Could anything be more absurd? There should be less red tape and more latitude in the purchase of comforts for the sick. A sufficiency of transports should be always available for the sick and wounded. The orderlies should receive higher pay and be recruited more carefully. The sanitary arrangements should be more directly under control of the medical department.

These are a few of the changes which will enable the medical department to more thoroughly and efficiently carry out its work of mercy and relief. The Royal Army Medical Corps contains some of the ablest and most capable men I have ever met. It is a credit to the army and an honor to the nation.—G. STERLING REYERSON, M.D., L.R.C.S. (Edin.), in *Canadian Practitioner and Review.*

---

***NOTICE.—This JOURNAL and THE INTERNATIONAL JOURNAL OF SURGERY one year for $1.00 cash, to new subscribers. No out-of-town checks accepted unless 10c. is added for cost of cashing. Strictly to new subscribers.***

### SOME OF THE DISEASES AFFECTING THE FEMALE BREAST FOLLOWING CONFINEMENT, AND TREATMENT.*

Some of the diseases which affect the female breast following confinement and during the early period of lactation are to the practitioner perplexing and at times annoying, because there is so little of importance in our authorities, as a guide in their treatment; and, as is usual, the minor points, which to us are of so much importance, are either left out entirely, or are treated so slightly that we are no wiser after perusal of all that can be found upon this subject. True, some writers treat some one condition very thoroughly and minutely, while they deal with others in a general way; but I have not yet found any authority treating the whole list of these maladies thoroughly.

It is not the intention of this paper to treat all the diseases which may occur during lactation, but only those with which I have had most to deal. Neither do I intend to go into detailed pathologic conditions, causes, or symptomatology except sufficiently to relate the condition present and the methods employed for their cure with gratifying results to the patients and myself.

Short or retracted nipples are more frequently met with in primipara than in multipara, and may be congenital in either case; but usually when encountered in the latter it is the result of cicatricial contractions from badly ulcerated nipples, inflammations, burns and neoplasms.

Treatment.—This condition can be benefited, if seen during pregnancy, by directing the patient to massage and draw the nipples daily. But it is not advisable to recommend such measures until after the middle of pregnancy, owing to the sympathetic condition that exists between the breasts and uterus. Another method I have employed is to take a test-tube large enough to encircle the nipple, then exhaust the air in the tube by burning a few drops of alcohol, and apply as you would dry cups. All these methods may seem to have done little in relieving the condition, yet from the handling and manipulations you have toughened the epithelium of the nipple and better fitted it for use, such as it may be.

*Read By Wm. B. Murphy, M.D., Minneapolis, before the Medical Club, Minneapolis.

Inflammation of the nipples is especially liable to occur during the early part of lactation; and in poorly nourished mothers in whom the nipples are not well developed, the child, while in the act of nursing, macerates and loosens the epidermis, which separating leaves a raw surface. This may deepen into a fissure or may ulcerate—thereby opening an avenue for infection, causing acute inflammation of the breast, with all its symptoms. The pain is increased with every attempt at nursing, hence the mother, from dread of it, lengthens the intervals, when the gland becomes distended and turgid with milk, causing elevation of temperature, chills and much anxiety. Sore nipples may be divided into—

1. Swollen and edematous.
2. Complete erosion of the top.
3. Fissures radiating in any direction.
4. Fissures at the base.
5. Small ulceration on some of the papillæ.
6. Ulcerations in the areola.

For the first of these conditions, thorough cleaning with soap and water, followed by antiseptic compresses of boric acid, borolyptol, or lysol, may be depended upon, the nipples being thoroughly dried and dusted with boracic acid, with lengthening of the period of nursing, thereby giving the nipples and breast as much rest as possible. In the other condition, where there are erosions, ulcerations, and small sacs of purulent or cystic material, I puncture with an aseptic needle or scalpel, and thoroughly wash with pure peroxide of hydrogen and some mild antisptic solution, such as boric acid or borolyptol. Then I paint once a day with a solution of nitrate of silver, grains 20 to the ounce, painting the nipple and areola and allowing it to dry. Then put on a sterile cotton pad, when necessary to nurse. I have the patient use a glass nipple shield with a rubber nipple attached, which prevents any further maceration of the epidermis, and keeps the nipple and areola as dry as possible, and promotes repair. After the child has finished nursing I instruct the nurse to wash the nipple and areola thoroughly with a hot boric acid solution, dry, and put on a sterile cotton pad. I find that these means will improve the conditions in from three to five days. The nipple shields should be scalded and placed in an antiseptic solution until needed again, keeping them entirely immersed and sweet and clean.

Acute mammitis may occur at any period of life, but usually occurs in nursing women during the first week of lactation. Its cause may be from infection, injuries, or the inability of the child to properly nurse and completely empty the breast, leaving it more or less distended with milk, which causes it to become swollen, tender and painful. If prompt measures are not used an abscess may soon form. This may be averted, if seen early, by at once giving an enema or good saline, with hot applications, or the reverse. Whichever is used, must be either as hot as can be borne or ice cold; and in using the latter, it must be seen to that the ice is in contact with the rubber and breast, and not floating around in the water its melting has formed. With these should be used gentle massage, which is produced by supporting the breast with one hand and using the tips of the fingers of the other hand, gently but firmly rubbing from the base toward the nipple until a free flow of milk is established, thereby completely emptying the breast. One must always be careful that no distended ducts remain.

Another condition closely associated with this is where the breasts are distended with milk so that the child is unable to nurse, because it cannot start the flow. The massage, as above described, loosens the distention, so that when the child is put to the breast the flow of milk is easy and natural; and the child is able to nurse completely, and to its satisfaction. Where the breasts show a tendency to fill quickly and remain filled, it is a good plan to have a tight-fitting vest with an opening for the nipples, or a wide or roller bandage to compress the breasts, thus preventing them from filling too rapidly.

I know of no more troublesome condition to contend with in this period than an abscess, because you not only have the breast to deal with, but the question of the proper nourishment for the child, and, if this occurs in the summer months, the condition is still more perplexing. The abscess should be promptly opened antiseptically, and the incision should always be radiating from nipple and areola in a line with the milk ducts, which can be both seen and felt, not transversely, because in this way you cut ducts that are not at all affected, and cripple them for life. It would seem unnecessary to emphasize this procedure, but I have seen it done contrary to this often repeated caution. The incision should be large enough to admit the finger, which is introduced to gently break up all pockets of pus, the wound being washed out with ($H_2O$) peroxide of hydrogen and sterile or normal salt water. I then take tr. iodine on an applicator, and thoroughly swab the cavity, rewash, and then pack loosely with a strip of iodoform gauze. I then use strips of adhesive plaster to firmly bind the breast to the chest, drawing the gland upward and inward, leaving an opening over the incision and the nipple. I then put on the usual gauze and cotton dressings retained with a roller bandage. The dressings are changed once or twice a day, as the case requires, the cavity being merely kept open by a piece of gauze in the skin opening and irrigated gently if there is much discharge. There is usually more or less milk coming from the nipple when the child nurses from the well breast (as the one affected is not used.) For this I arrange the dressings so that a pad of cotton may be kept over the nipple and frequently changed, thereby keeping the other dressing free from contamination with the milk. By this method I find that the abscess cavity heals in from three to four weeks, while by the old methods it took two or three months, besides the annoyance of a slipping bandage, and the saturation of bandages and dressing with milk and secretions from the abscess. When the abscess is about healed, a milk fistula may prolong recovery, but a few applications of nitric acid and a little pressure will soon cure this. During the recovery from this condition the general health of the mother must be kept up by fresh air, rest, and general tonics.

Now, what is the after result of these cases? In one case I saw, where the ducts were severed transversely, nursing was resumed without any seeming difficulty, but what the result will be at future pregnancies I am not able to state. In another case treated as above, nursing was reestablished without any trouble. In another at a subsequent lactation the breast did not take on its natural function and everything was done to establish the flow of milk, but only a small quantity would be secured, and all the while there was much pain and tenderness at the old scar, and engorgement in the ducts severed centrally to the scar.* Toward the latter months of nursing the pain and soreness left, but the breast was crippled. Of the cases I relate, some were those of midwives and some of other physicians. One case of abscess occurred seven months after delivery, with no abrasion of the external surface, but the patient had sore nipples in the early part of lactation.—*North Western Lancet.*

---

### MUSIC.

Music is a neglected therapeutic agent. Like some good doctors, it does not get the credit it deserves. Amongst the thousands of medical schools in the United States it would be hard to find a single text-book on music.

They have a custom in Germany which could be profitably borrowed by those who study there. Anything like a diamond ring or a steel rail cannot be borrowed from Europe. The custom house is an object-lesson on honesty to tourists. A custom can be smuggled, thus illustrating the value of education abroad. The daughter of a high-toned pig-sticker or coal-oil barron can go to Holstein, Jersey or Durham, and import a blooded husband with impunity. The administration, if it was not controlled by trusts, and if it really was in earnest about protecting our infant industries, would either levy an *ad valorem* duty, or better, confiscate the husband.

German youth who go to a musician to have their talent refracted are told how Dr. So-and-So's medicine causes a hot-house development of genius; and *vice versa,* little princesses are advised to take two hours systematic exercise of the upper extremities to develop the bust. As they pass out of the reception room they are handed a card which reads:

PROF. VON BUNG,
Bustificator for the Royal Family.

Away back toward the front end of the Athenian Encyclopedia of Practice, issued 237 B. C., Hippocrates has a scholarly and acute

---

* This case was not treated by me when she had the abscess.

monograph on the origin of the title M. D. The article as a whole makes me feel certain the founder of medicine was a college-bred man. His portrait, which accompanies the article, gives us the idea that old Hip. had a prodigious cerebral measurement and a double crown. He wears several buttons which are probably badges of Greek letter fraternities.

The body of the work is embellished with crude wood-cuts, such as are furnished by our up-to-date contemporaries of to-day. They remind the busy worker of the first draft of a mental concept. In this case they represent the physician with a lyre and an obstetric bag, shaped something like a pair of suspenders. His attitude in one place indicates that he is practicing medicine on a primipara; and in a second he seems to be trying to collect his bill. We can recognize the primipara in the second picture, although there has been pronounced growth of the maternal instinct. The laity scattered about the background are expressing by signs the unanimous opinion that the doctor charged too much, while he points to his lyre, as if to call the attention of the crowd to its value as an oxytocic. Hippocrates makes an effort to classify the musical knowledge of the period. Roughly speaking, flats were applicable to women and sharps to men. Chords of the minor fifth were supposed to be specific for the gonococcus.

Italy was settled by Greeks, who believed in expansion. When the Italians swarmed in America they carried with them their love for macaroni and an inherited bias for imperialism. McKinley contracted the disease by purchasing a sack of hot roasted peanuts from the Italian ambassador in Washington. Writing prescriptions in the dead languages was also acquired from Italy. Lately a reaction toward shorthand has set in for prescription work. It is argued by the reformers that the new system will have an elevating effect on the moral tone of the drug store.

About the time the cargo of tea arrived in Boston, in 1775, an old Yankee doctor had commenced to advocate the theory that the teapot was harder on nerves than the whisky bottle. He was a man who was not afraid to ride on the cowcatcher of public opinion, and besides being a violin virtuoso he could bewitch people, as they called hypnotism then, and claimed to understand just what the renaissance meant. It was his boast that he used common sense instead of science in his practice with children. Rather than take a postgraduate course in child-study, as was the fad at that time, the sly rascal took a vacation and got married. In the course of time he hitched up the spring wagon and sent his wife to the mountains for her health, while he took care of the baby. If there was anything the baby did not do that summer the doctor said he could not find it mentioned in the bibliography of the subject.

Patriotism flamed up in Boston. The people destroyed the tea and then sent word to King George that the doctor was to blame. They were insincere, however, as is proved by the fact of everybody having boiled harbor water for breakfast the next morning.

Benjamin Franklin eased the strain on the continental conscience in the constitutional convention of 1787, after the three great compromises of our charter of liberties, by playing "Home, Sweet Home," on the mouth organ. Mr. Franklin was a philosopher, but he failed to reflect on the possibility of his example making this little musical go-devil popular to the third and fourth generation. How often has it been observed since the Revolutionary epoch that a boy can make as much noise in the daytime as a cat can at night.

Music inspires men to commit heroic depredations of valor. Likewise many weak-legged women have been snatched from an untimely grave by taking dancing lessons.

In Puritan New England, which has furnished most of our literature, as well as some history, it was obligatory to give one-tenth to the church. Physicians got into the habit of letting the deadbeats die and crediting their accounts to the cause. The system had its advantages, for when an honest man fell sick he would invariably pay in advance, or at least give his note. Fear of the Lord and the doctor is always the beginning of wisdom.

One of the most striking clinical facts of the century was lately recorded in New Jersey. A music teacher married a man who had his life insured for $10,000. Very soon it was noticed the mosquitoes would not bite the wife so long as the husband was there to furnish bites. In the course of a few years he contracted malaria and gradually died off. Everything was done for the poor man the profession could think of. As a last resort the quinine bottle was set on the table at meal times, and he was encouraged to help himself. The wife allowed herself to be sued by the insurance people to compel acceptance of their check for the full amount of the policy. Occasionally we meet up with a woman who is conscientious instead of contrary.

Down in Indiana last March a man from Illinois was delivering samples to the medical men of Cherubusco. In an office he found a culture of Eberth's bacillus sitting on the window-sill, which he supposed was a drink of whisky. At Knoxville, Tennessee, he developed a typical case of typhoid fever. His nurse was a frigid, domineering woman, more fit to keep house than to make a man comfortable. The patient threshed around for several days, abusing the climate and poking sarcasm at the southern ideal of womanhood, and finally called for a brass band. To the nurse's surprise, defervescence occurred, accompanied by profuse diaphoresis. The heart rallied, the tongue cleaned off, the appetite returned, and the patient fell into a sound and refreshing sleep. Next day the nurse telephoned the medical attendant, saying:

"Hello, Doc! Come over here right away and keep this damn fool from getting out of bed."

The doctor forthwith shut himself up in his intellectual apparatus and wrote a seventeen-column article for publication on the application of modern methods to private practice.

Our profession is full of good singers, but medical editors tell me a physician with a cultivated ear is usually in arrears. Very few periodicals are endowed with anything more than a brainy editor, and the stern chase after the almighty dollar compels the man at the helm to lay the most brilliant and original piece of work on the table, until hard times subside in the authors' section. Wide-awake men do not write as much for money as they do for publication. A persistent cultivator of a medical style has ethical motives. He knows his article will cause an epidemic of jealousy amongst his fellow-practitioners, besides being a stimulus to the orderly arrangement of thought, good penmanship and grammar, the use of a medical dictionary and one side of the paper.

Luxuriant crops of experience allowed to go to seed will cause mental rickets. The things a man forgets are usually what he needs to give his reputation a new coat of paint.—*James E. Free, M.D., Billings, Montana, in Denver Medical Times.*

---

### THE PSYCHOSES OF PUBERTY.

PROFESSOR LICHEN, JENA.

As a result of the study of four hundred cases of insanity, the first appearances of which took place at the time of puberty—that is fifteen to twenty-one years of age—I have come to the following conclusions:

1. The morbidity curve of the psychosis shows its maximum in the time of puberty. Puberty psychoses are found more frequently in those with a neuropathic heredity than those without. In addition to the neuropathic heredity, chlorosis, physical and mental exhaustion, sexual excesses, and acute infectious diseases are of pathological especial importance.

2. Almost all varieties sf psychoses are found in the age of puberty. The latter exercises a special influence in so far only that it favors the appearance of certain psychoses and often, not always, modifies them in symptoms and in course in a definite manner (hebephrenic modification). The assumption of a special puberty psychosis which shall include the great majority of the psychoses of puberty is unwarranted. The only psychosis which occurs almost exclusively at the age of puberty is hebephrenic dementia or kahlbaunis hebephrenia; but under this category is included only a relatively small number of the psychoses of puberty.

3. The psychoses which occur often in the puberty period outside of hebephrenia are mania, melancholia, circular insanity, acute hallucinatory paranoia (amentia of many authors), and certain hysterical and epileptic psychoses.

4. The most important modifications which puberty causes are the following: Increased instability of affective disturbances, incoherence of the normal succession of ideas, delusions, tendency to mimic, verbal, etc., stereotype, illogical, foolish, fantastic content of delusions, and a tendency to the circular forms on the one hand and to progressive dementia on the other. All these modifications are found more often in puberty psychosis than in others.

5. The prognosis in general is more earnest and especially in regard to the last mentioned modifications.

6. The treatment is much the same as in the like psychoses of adult life. Complete rest in bed, however, with the exception of cases of great exhaustion, is not advisable. Of great importance in most cases is a regular, carefully thought-out employment of body and mind.

The use of narcotics should be as far as possible avoided. Being together with adult insane patients is to be allowed only with great precaution and selection.—*St. Louis Med. Review.*

---

### THE USE OF HYDROZONE AND GLYCOZONE IN GASTRIC AND INTESTINAL DISTURBANCES.

I have, for a long time, been rather enthusiastic over the value of hydrozone and glycozone in treating diseases, and can attribute much valuable assistance and extraordinary results from their use in the last few years. The medical profession, in fact, has never gained such remarkable results from the employment of any production as it has from the use of these preparations, and my recent effects have almost, in a measure, surpassed them all. I will give a brief report of one remarkable case. I could mention several others, but a physician's time is valuable, and often he has not the moment to spend in perusing a legion of cases, so I select this one, it being the severest of all, to demonstrate, the potency of hydrozone and glycozone:

I was called to treat a young man, suffering from a severe gastroenteritis. I found him in a most serious condition, having been delirious for three days. His temperature was subnormal, 97.6, pulse 60, respiration 16. He was greatly emaciated, atonic, had inappetence, a severe agonizing pain in the stomach and intestines, at times so severe that he would sit on the edge of the bed and groan, oftentimes yell. These attacks were always of a similar nature and occurred regularly. He was unable to take either solid or liquid food, even in small quantities without causing a return of the pain, a teaspoonful of milk being sufficient to produce it. His condition was pitiable. His cheeks were hollow, eyes congested, skin pale and sallow, and his whole appearance showed the presence of intense pain.

I was called at the end of the third week of his illness. The former physician had employed opiates in large doses with most worthless results, also many other drugs with not a sign of improvement, he growing seriously worse. I determined that hydrozone and glycozone were the remedies indicated, and were the only ones that would be of value here, therefore, I gave him, at once, one-half glass of a mixture of one-half ounce of hydrozone with a little honey to one quart of water. He was somewhat disturbed for a while after the portion, but was soon relieved. The distress, I presume, was due to the advanced stage of the inflammation. I continued to administer this for some time, with only a slight improvement, but after several doses had been taken, the relief was very decided. After his nourishment, I gave one teaspoonful of glycozone in a wine-glass of water. After a few doses of this, he was much easier, and at midnight fell asleep and slept all night, not awakening until morning, the first sleep that he had had in five days. I had previously discarded all other remedies, of which there was a large number, as one after another was given with no benefit. All of the acute symptoms disappeared in a few days, at which time he felt very much better, and he continued to improve without having a recurrence of any of his old severe symptoms. Before this, I had increased both the nature and the quantity of his food, which he relished greatly. I continued the hydrozone and glycozone for a month after, to entirely reduce the inflamed condition of the mucous membrane of the gastro-intestinal tract. These two remedies have afforded me most excellent issues many times in the treatment of gastric and intestinal disorders.

All gastric and intestinal disturbances are caused by the lining of the stomach becoming inflamed, and in order to allay this inflammation it must first be treated with antiseptics, then with medicaments, that both heal and stimulate the mucous membrane that has become diseased. The most common cause for this state of inflammation is a greatly diminished quantity of gastric juices necessary for digestion, consequently, the food partaken of, instead of being assimilated, ferments; in other words, the peptic glands, whose function it is to secrete the gastric juice, do not perform their function properly. These must be restored to their normal state at once, which is accomplished by remedies that exert a stimulating effect upon them, and at the same time are non-toxic, else the trouble will only be aggravated. Hydrozone and glycozone are the two remedies par excellence for these two purposes, and the success that I have obtained from the employment of them during the past few years will lead me to always use them in these disorders.

Hydrozone causes destruction to microbes, has no deleterious action upon animal cells, possesses no toxic qualities, exerts no corrosive effect upon healthy mucous membranes when used in diseases caused by germs, is a pus-destroyer and a stimulant to granulating tissues. Hydrozone is destruction itself to the skin or mucous membrane that has become diseased, and leaves the subcutaneous tissues in a perfectly healthy state.

Glycozone, while not so rapid in its action as hydrozone, is, nevertheless, just as sure a stimulant, and in all gastric and intestinal disorders exerts a potent and uninjurious effect upon the diseased mucous membrane of the stomach, healing it to a nicety. It is an effective oxidizing agent, has an agreeable, sweet and, at the same time, slightly acid taste resembling lemonade. Its use produces no deleterious action on the heart, liver or kidneys.

The beneficial results which hydrozone and glycozone have afforded me in the treatment of this class of disorders have caused me to discard all the other methods of treatment by drugs that exert an ephemeral influence, but do not jugulate the offending condition. What is needed in these diseases is an antiseptic that will destroy all pathogenic germs, and at the same time stimulate the walls of the stomach. Hydrozone kills the bacteria, dissolves the mucus and prepares the stomach to better digest the food; in short, it deterges the stomach, hence in it we have an efficient antiseptic; glycozone removes the mucous from the walls of the stomach, stimulates and heals. I have discovered these two preparations to be ideal ones in treating this very common and distressing disorder.— *W. H. Vail, M.D., Physician and Surgeon, St. Louis, Mo., in Medical Mirror.*

